# Elongation of the disc-fovea distance and retinal vessel straightening in high myopia in a 10-year follow-up of the Beijing eye study

**DOI:** 10.1038/s41598-021-88579-9

**Published:** 2021-04-26

**Authors:** Rahul A. Jonas, Yan Ni Yan, Qi Zhang, Ya Xing Wang, Jost B. Jonas

**Affiliations:** 1 Beijing Institute of Ophthalmology, Beijing Tongren Eye Center, Beijing Tongren Hospital, Capital Medical University, Beijing Ophthalmology and Visual Science Key Laboratory, Beijing, China; 2 Department of Ophthalmology, University Hospital of Cologne, Cologne, Germany; 3 Department of Ophthalmology, Beijing Tongren Eye Center, Beijing Tongren Hospital, Capital Medical University, Beijing Ophthalmology and Visual Science Key Laboratory, Beijing, China; 4 Eye Center, The 2nd Affiliated Hospital, Medical College of Zhejiang University, Hangzhou, China; 5 Department of Ophthalmology, Medical Faculty Mannheim, Heidelberg University, Mannheim, Germany; 6 Institute of Molecular and Clinical Ophthalmology, Basel, Switzerland

**Keywords:** Diseases, Eye diseases, Macular degeneration

## Abstract

To assess changes in the disc-fovea distance (DFD) in highly myopic eyes in a 10-year population-based follow-up study. The case control study included all highly myopic eyes (myopic refractive error ≥ − 6.0 diopters or axial length ≥ 26.0 mm) and a randomized group of non-highly myopic eyes examined in the population-based Beijing Eye Study 2001 and 2011. Using fundus photographs and optical coherence tomographic images, we assessed changes in DFD, parapapillary gamma zone, angle kappa (angle between the temporal arterial arcades), and course of papillo-macular retinal vessels. The study included 89 highly myopic eyes and 86 non-highly myopic eyes. DFD elongation, gamma zone widening, angle kappa decrease and straightening of papillo-macular retinal vessels were detected more often (all *P* < 0.001) in the highly myopic group than in the non-highly myopic group (63/89 versus 9/86;75/89 versus 18/86;61/89 versus 9/86; and 58/89 versus 7/86,respectively). Gamma zone enlargement, angle kappa reduction and papillo-macular retinal vessel straightening were significantly (all *P* < 0.001) associated with DFD elongation. The length of macular Bruch’s membrane on the disc-fovea line and the vertical distance between the temporal arterial arcade did not change during follow-up. DFD elongation (10-year incidence 70.8% in highly myopic eyes) was associated with gamma zone enlargement, while macular Bruch’s membrane length remained unchanged. It supports the notion of a temporal shift of an otherwise stable posterior Bruch’s membrane in axially elongated eyes. Straightening of the papillo-macular vessels with increasing gamma zone width suggests a coincident stretching of the papillo-macular retinal nerve fibers and inner limiting membrane.

## Introduction

Within the optic nerve head canal, three layers can be differentiated: Bruch’s membrane opening (BMO), the opening in the choroidal layer delineated by the peripapillary border tissue of the choroid, and the opening in the scleral flange, covered by the lamina cribrosa and delineated by the peripapillary border tissue of the scleral flange^1–3^. Recent studies have suggested that the process of axial elongation in moderately myopic eyes is associated with a backward shift of the BMO, usually in direction towards the fovea^4,5^. Since the two deeper layers of the optic nerve head canal, i.e. the choroidal opening and the peripapillary scleral flange opening, do not fully follow this movement, the backward shift of the BMO leads to an overhanging of BM into the intrapapillary compartment at the nasal disc border, and a lack of BM in the temporal parapapillary region^6^. A parapapillary region without BM has been termed gamma zone^5,7–12^. Presence and size of gamma zone in the temporal region is associated with a longer disc-fovea distance (DFD) while the length of the macular BM, measured as distance between the fovea and the temporal gamma zone border, is independent of axial length^10^. In a parallel manner, the fovea is located more inferiorly in eyes with an inferior gamma zone than in eyes with a superior gamma zone^13^.

These observations made in cross-sectional studies led to the notion that BM with its associated retinal pigment epithelium and photoreceptor layer may shift during the process of axial elongation into the posterior direction, potentially caused by a formation of new BM in the equatorial region^14^. To further assess that hypothesis, we examined in the present follow-up study longitudinal associations between changes in the DFD and changes in the parapapillary gamma zone. To avoid a potential bias by the referral of patients to a hospital, we choose a population-based recruitment of the study population.

## Methods

The Beijing Eye Study is a population-based longitudinal study performed in a rural region and in an urban area of Greater Beijing^15^. The Medical Ethics Committee of Beijing Tongren Hospital approved the study protocol according to the declaration of Helsinki and all study participants gave their written informed consent. The ethics committee confirmed that all methods were performed in accordance with the relevant guidelines and regulations. First conducted in 2001, the survey was repeated in 2011 inviting all participants from the survey of 2001. Included into the present study were all those individuals who had participated in the surveys in 2001 and in 2011 and who were highly myopic, defined by an axial length of ≥ 26.0 mm or if axial length measurements were not present, defined by a myopic refractive error of more than − 6 diopters in 2001 or 2011.

As also described in detail previously, all participants underwent a structured questionnaire, systemic examinations, and a comprehensive ophthalmic examination^16^. The latter included measurements of visual acuity, slit lamp examination of the anterior and posterior segment of the eye, and photography of the cornea, lens, optic disc and macula (fundus camera Type CR6-45NM; Canon Inc., Tokyo, Japan). In 2011, the optic nerve head including the peripapillary area and the macula were additionally imaged by spectral-domain optical coherence tomography (OCT) (enhanced depth imaging mode) (Spectralis, Heidelberg Engineering Co, Heidelberg, Germany). The protocol for imaging the optic disc and the macula included six radial scan lines with a scan length of 6 mm, centered on the optic disc and macula, respectively, and each comprising 100 A-scans. The parapapillary region was examined with the intrinsic viewer (Heidelberg Eye Explorer software version 1.7.0.0; Heidelberg Engineering), which automatically synchronized the vertical lines of each B-scan and the infrared image taken by the OCT device. The parapapillary gamma zone was defined as the region between the end of BM and the border of the optic disc^16^. The optic disc border was defined as the end of the lamina cribrosa or as the inner side of the peripapillary border tissue of the peripapillary scleral flange. The OCT image of the macula served to determine the localization of the foveola, and the optic nerve head imaging served for defining the border of gamma zone.

**Figure 1:**
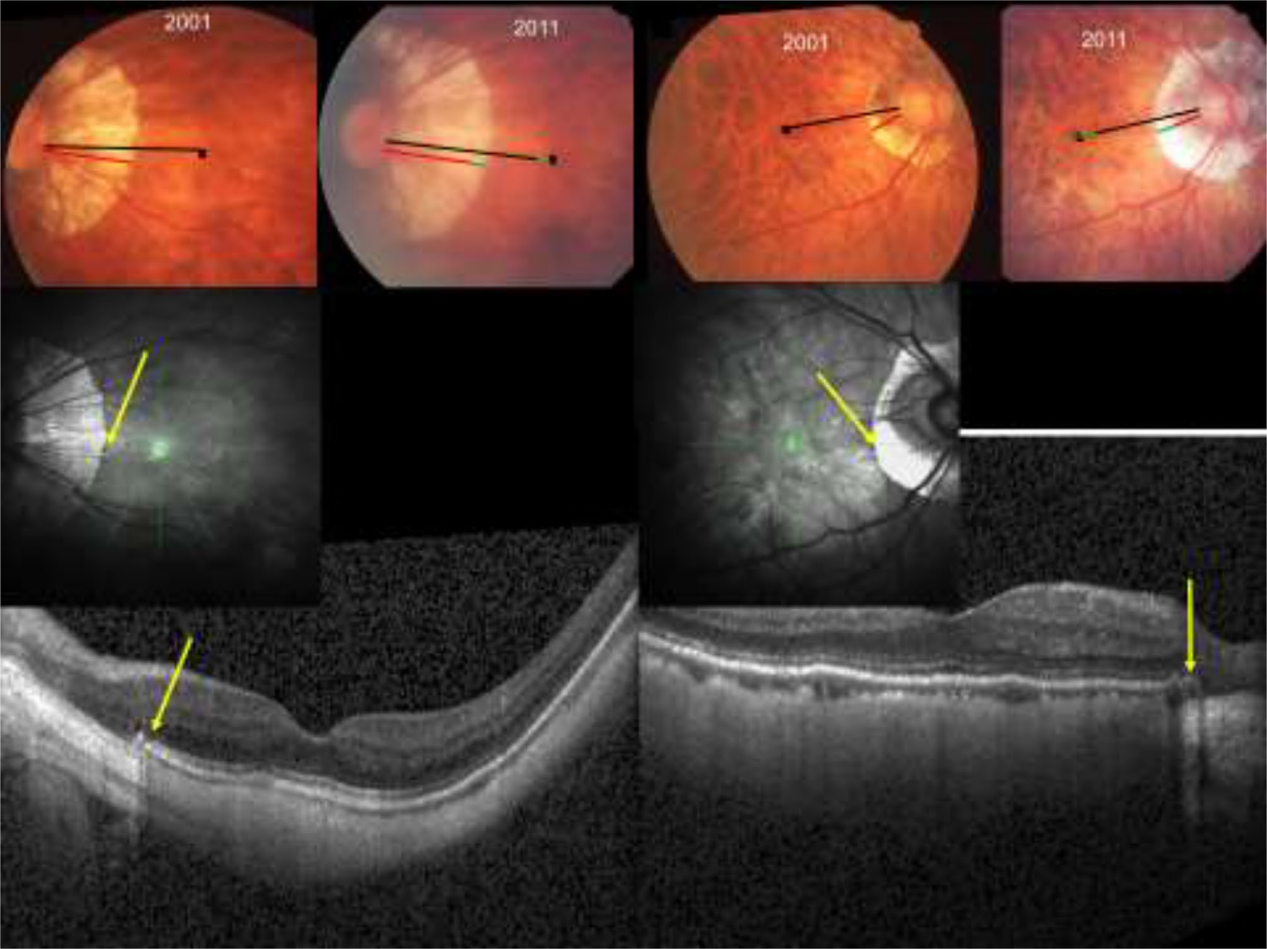
Upper part: Fundus photographs of two highly myopic eyes examined in 2001 and in 2011, showing an elongation of the disc-fovea distance (black bar, as measured in 2001; green bar: elongation in 2011) and the enlargement of gamma zone (red bar, as measured in 2001; green bar: amount of elongation of the disc-fovea distance); black cross: foveola. Lower part: Optical coherence tomographic images showing the end of Bruch´s membrane (yellow arrows) indicating the border of parapapillary gamma zone.

**Figure 2:**
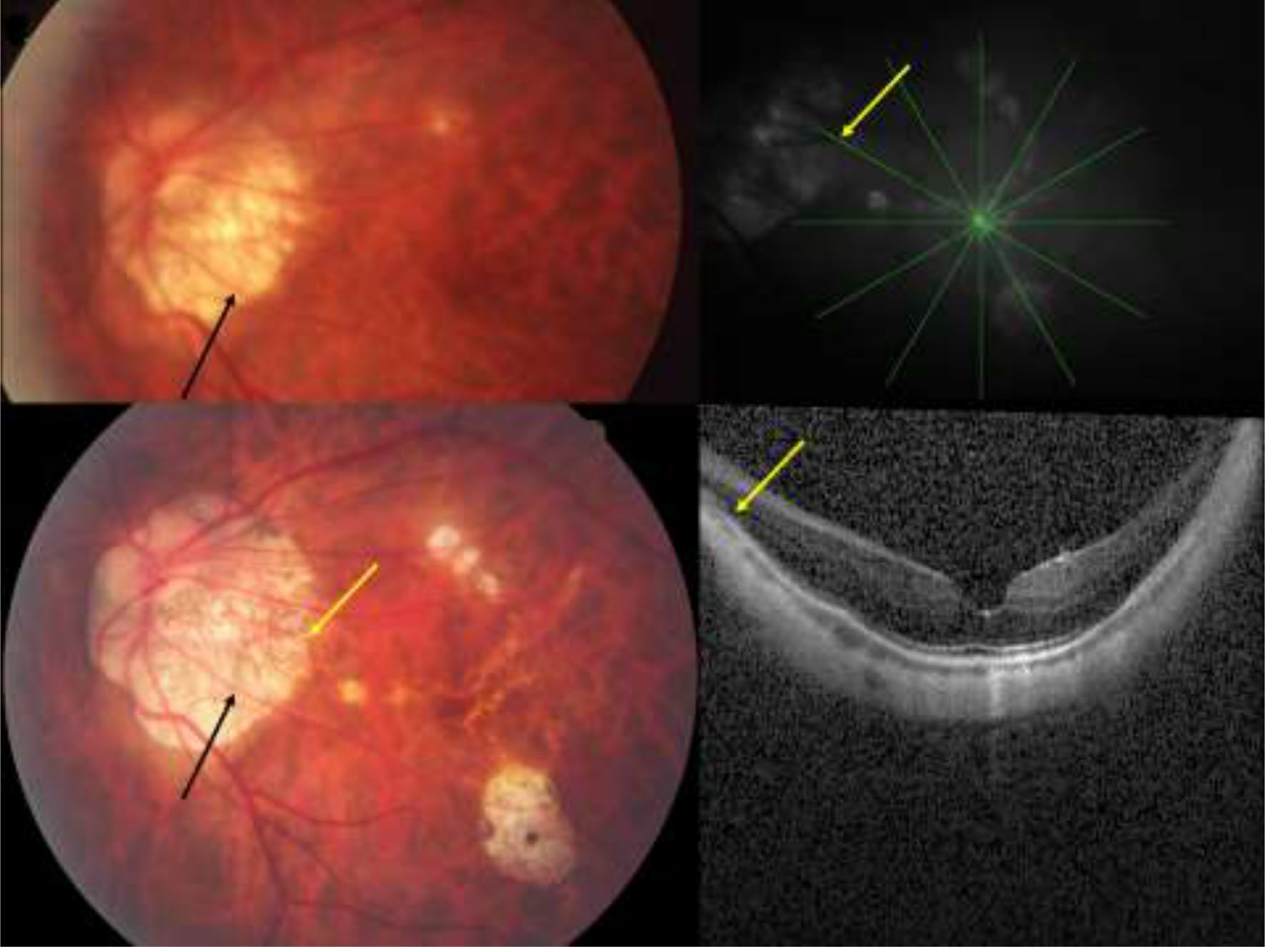
Left part: Fundus image of a highly myopic eye, taken in 2001 and in 2011 and showing the straightening of the retinal vessel (black arrow) in association with a widening of parapapillary gamma zone and an elongation of the disc-fovea distance in a highly myopic eye. Right part: Optical coherence tomographic images showing the end of Bruch´s membrane (yellow arrows, also on the lower left image) indicating the border of parapapillary gamma zone.

On the fundus photographs, we defined gamma zone as the parapapillary region, in which the sclera was clearly visible and covered by superficial retinal tissue (i.e. retinal vessels and retinal nerve fibers) while the choroid, except for few large choroidal vessels, was completely absent (Fig. 1)^7,17^. The delineation of gamma zone on the fundus photographs was controlled by marking the border of gamma zone on the OCT images. The DFD was assessed as the distance between the foveola and the optic disc center (Fig. 1)^10^. The position of the foveola was manually identified on the fundus images with the help of OCT images. The angle kappa was defined as the angle between the crossing point of the temporal superior arterial arcade with a vertical line drawn through the fovea, the optic disc center, and the crossing point of the temporal inferior arterial arcade with a vertical fovea line^18^. The length of the macular BM on the disc-fovea line was defined as the distance between the foveola and the border of gamma zone. The vertical distance between the temporal superior and temporal inferior temporal arterial arcade was the distance between both arterial arcades measured on a vertical line running through the fovea. Presence and stage of myopic macular degeneration were specified according to the recommendations made by the Pathologic Myopia Study Group^19^. Lacquer cracks were considered as part of category 3 of myopic macular degeneration. Aligning and flickering the photographs of the optic disc and macula, taken in 2001 and 2011, we assessed and noted any changes in the DFD, in the presence, size and location of parapapillary gamma zone, in angle kappa, and in the course of the papillomacular retinal vessel. Retinal vessel straightening was defined as a more straight, less contorted, course of a retinal vessel in the papillomacular region (Fig. 2).

Using a commercially available statistical software package (SPSS for Windows, version 25.0, IBM-SPSS, Chicago, IL, USA), we first calculated the mean and standard deviations of the demographic parameters. Using the chi-square test, we assessed associations between the occurrence of changes in the DFD and the occurrence of changes in gamma zone, changes in angle kappa, and the course of the retinal vessels in the temporal region. All *P*-values were two-sided and considered statistically significant when they were < 0.05.

## Results

Out of 4439 individuals originally participating in the survey in 2001, 379 individuals had died and 1365 individuals had moved or did not want to be re-examined in 2011, so that the survey in 2011 included 2695 individuals who had also participated in the survey of 2001. They had a mean age of 64.7 ± 9.7 years (range 50–93 years) in 2011 and a mean refractive error of − 0.20 ± 2.13 diopters (range − 22.0 to + 7.5 diopters). Out of 204 highly myopic eyes with a myopic refractive error of ≥ − 6.0 diopters and/or an axial length of more than 26.0 mm in the survey of 2001, 89 eyes (61 participants; 24 (39.3%) men) were re-examined in the survey of 2011 and had assessable fundus photographs from both surveys. The age of these participants in 2011 (65.0 ± 9.8 years; median: 66 years; range 50–88 years) did not differ significantly (*P* = 0.17) from the age of the remaining participants in the survey of 2011 (64.7 ± 19.7 years (median: 64 years; range 50–83 years). The mean axial length was 27.6 ± 1.3 mm (range 26.06–30.88 mm). Cataract surgery had been performed in 11 out of the 89 eyes. For the remaining eyes without cataract surgery, the mean refractive error was − 9.41 ± 3.52 diopters in 2001 and − 9.43 ± 3.26 diopters in 2011 (Table 1).

**Table 1:** Demographic and ocular characteristics and comparisons between the highly myopic group and the control group.

	Highly myopic group	Non-highly group	*P*-value
n (eyes/participants)	89/61	86/57	
Age (years)	65.0 ± 9.8	66.7 ± 9.7	0.25
Gender (women)	37 (60.7%)	36/57	0.89
Refractive error (spherical equivalent) in 2001 (diopters)	− 9.41 ± 3.52	− 0.97 ± 2.61	< 0.001
Refractive error (spherical equivalent) in 2011 (diopters)	− 9.43 ± 3.26	− 1.02 ± 2.45	< 0.001
Axial length (mm) in 2011	27.6 ± 1.3	23.6 ± 1.1	< 0.001
Myopic maculopathy grading in 2001 (Category 0/1/2/3/4)	11/58/10/8/2	73/13/0/0/0	< 0.001
Myopic maculopathy grading in 2011 (Category 0/1/2/3/4)	9/38/25/13/4	72/14/0/0/0	< 0.001
Disc-fovea distance elongation	63/89 (70.8%)	9/86 (10.5%)	< 0.001
Gamma zone widening	75/89 /84.3%)	18/86 (20.9%)	< 0.001
Angle kappa decrease	61/80 (68.5%)	9/86 (10.5%)	< 0.001
Papillo-macular retinal vessel straightening	58/89 (65.2)	7/86 (8.1%)	< 0.001

The present study additionally included a group of 86 randomly selected eyes (57 individuals) out of the non-highly myopic group who had been examined in both surveys in 2001 and 2011. Mean refractive error in 2001 and in 2011 was − 0.97 ± 2.61 diopters and − 1.02 ± 2.45 diopters, respectively (Table 1). The mean axial length was 23.6 ± 1.1 mm (range 21.64–25.90 mm). The highly myopic group and the non-highly myopic group did not differ significantly in age (65.0 ± 9.8 years versus 66.7 ± 9.7 years (median: 68 years; range 50–84 years: *P* = 0.25) and gender (*P* = 0.89).

Within the highly myopic group, 42 (48%) of the 89 highly myopic eyes showed a progression of myopic maculopathy. Progression was observed in two (18.2%) out of 11 eyes with no fundus tessellation, 24 (41.4%) of 51 eyes with tessellated fundus (category 1) at the initial examination, in 6 (60%) of 10 eyes with diffuse chorioretinal atrophy (category 2), in 8 (100%) of the 8 eyes with patchy chorioretinal atrophy (category 3), and in two (100%) out of two eyes with macular atrophy (category 4) in 2001. In 2011, 38 (42.7%) eyes were categorized as “tessellated fundus” (category 1), 25 (28.1%) eyes as “diffuse chorioretinal atrophy” (category 2), 13 (14.6%) eyes as “patchy chorioretinal atrophy” (category 3), and 4 (4.5%) eyes as “macular atrophy” (category 4).

The mean DFD was significantly longer in the highly myopic group than in the non-highly myopic group (5.74 ± 0.50 mm versus 4.87 ± 0.48 mm; *P* < 0.001). In the 89 highly myopic group, an elongation of the DFD was detected in 63 (70.8%) of the eyes, in 25 (28.1%) of the eyes the DFD remained constant, and in one (1.1%) eye, the fovea moved inferiorly. In the 86 non-highly myopic group, the DFD elongated in 9 (10.5%) eyes and became shorter in one eye (1.2%), while in 76 (88.4%) eyes, the DFD did not change. The difference in the prevalence of an elongation of the DFD between both groups was significant (*P* < 0.001). Within the non-highly myopic group, the subgroup of eyes with DFD elongation as compared to the eyes without DFD elongation was significantly more myopic (− 3.97 ± 3.92 diopters versus − 0.67 ± 1.98 diopters; *P* < 0.001).

A widening of parapapillary gamma zone was detected significantly more often (*P* < 0.001) in the highly myopic group (75/89 (84.3%) eyes) than in the non-highly myopic group (18/86 (20.9%) eyes). In the total study group, and in the non-highly myopic group and in the highly myopic group taken separately, the enlargement of gamma zone was significantly (all *P* < 0.001) associated with the elongation of the DFD. In the group of 93 eyes with a widening of gamma zone, 72 (77.4%) eyes showed a DFD elongation, while in the group 82 eyes without gamma zone widening, the DFD did not elongate in any eye.

In the highly myopic group, a decrease in angle kappa without evident cause was observed in 61 (68.5%) eyes, and angle kappa became smaller in association with a rotation of the temporal arterial arcade downward in two (2.2%) eyes. In the 86 non-highly myopic group, 9 (10.5%) eyes showed a decrease in angle kappa, one (1.2%) eye showed a decrease in angle kappa in association with the development of an epiretinal membrane, and in 75 (88.4%) eyes angle kappa remained unchanged. In the total study group, and in the non-highly myopic group and in the highly myopic group taken separately, the reduction in angle kappa was associated (all *P* < 0.001) with the DFD elongation.

The distance between the temporal superior arterial arcade and the temporal inferior arterial arcade assessed on a vertical line through the fovea did not change in any of the 93 highly myopic eyes without macular BM defects (i.e. category 2 or less of myopic maculopathy), except for one eye with a decrease in angle kappa in association with the development of an epiretinal membrane, and it did not change in any non-highly myopic eye.

A straightening of retinal vessels in the temporal sector was observed in 58 (65.2%) highly myopic eyes, while in 31 (34.8%) of the highly myopic eyes, the vessel course remained unchanged. In the non-highly myopic group, the vessel course remained unchanged in 77 (89.5%), while the vessels appeared to be straightened in 7 (8.1%) eyes and to be less straight in 2 (2.3%) eyes. The difference in the incidence of vessel straightening between both groups was significant (*P* < 0.001). Temporal vessel straightening was present (*P* < 0.001) in 64 of the (88.9%) 72 eyes with an DFD elongation and one (1.0%) of the 103 eyes without DFD elongation. In the total study group, and in the non-highly myopic group and in the highly myopic group taken separately, the retinal vessel straightening was associated (all *P* < 0.001) with the DFD elongation.

In multivariable analysis, the frequency of a DFD elongation was associated with a higher prevalence of an enlargement of gamma zone (*P* < 0.001), a more myopic refractive error (*P* = 0.03), and a higher prevalence of progression of myopic maculopathy (*P* < 0.001).

## Discussion

In this population-based sample of highly myopic eyes, the prevalence of a DFD elongation during a follow-up of 10 years was relatively high (70.8%), and it was associated with a widening of gamma zone, a decrease in angle kappa, and a straightening of the papillo-macular retinal vessels. In a multivariable analysis, a higher prevalence of a DFD elongation additionally depended on a more myopic refractive error and a higher prevalence of progression of myopic maculopathy. In all eyes without macular BM defects and without epiretinal membranes, the length of the macular BM on the disc-fovea line and the vertical distance between the temporal arterial arcades did not change during the study period.

The observations made in this longitudinal study agree with findings obtained in a recent cross-sectional investigation on a larger group of study participants in which a longer DFD was correlated with a wider gamma zone and a longer axial length^10^. In that study, the longer DVD in axially elongated eyes could fully be explained by the wider gamma zone, while the length of the macular BM on the disc-fovea line, measured as distance between the foveola and the border of gamma zone, was independent of axial length^10^. In a similar manner, the vertical distance between the temporal arterial arcades was not related with axial length if eyes with macular BM defects were excluded^18^. Similar findings were made in the present longitudinal investigation. It suggests that the widening of gamma zone is the cause of the DFD elongation, while BM in the macular region remained unaffected by axial elongation, unless macular BM defects developed. It leads to the notion that the macular region, outside of the parapapillary area with gamma zone and delta zones, remains primarily untouched by the process of axial elongation, except for an axial elongation-associated thinning of the choroid and a potential, axial elongation-associated stretching of the retinal nerve fibers in the papillo-macular bundle region (see below). The finding of a constancy in the macular BM dimensions is paralleled by observations made in histologic and clinical studies, which showed that the thickness of the retina, the density of the retinal pigment epithelium and the density of the choriocapillaris in the macular region were not related to axial length^20–23^. Correspondingly, best corrected visual acuity was not dependent on axial length if eyes with a myopic maculopathy were excluded^24^. It agrees with the notion that the distance of the macular photoreceptors was not enlarged in axially elongated eyes, in agreement with the histologic findings of an independence of the density of the retinal pigment epithelium and the choriocapillaris thickness of axial length. The decrease in angle kappa in eyes with a DFD elongation and a constancy of the vertical distance between the arterial arcades can geometrically be explained. The observations support the notion of a shifting, and otherwise stable, BM at the posterior pole in axially elongating eyes^18^.

Another finding of the present study was the straightening of the papillo-macular retinal vessels in eyes with an enlarging DFD and widening of gamma zone. The retinal nerve fibers and the inner limiting membrane are the only anatomical structures which connect the other retinal layers, including the retinal ganglion cell layer, with the optic disc. Both, the inner limiting membrane and the retinal nerve fibers are firmly connected to the optic nerve head. The lengthening of the distance between the macular ganglion cells and the optic disc by the enlarging gamma zone may thus lead to a straightening of the retinal nerve fibers, parallel to the straightening of the papillo-macular retinal vessels. If the retinal nerve fibers had a curved course to the optic disc before the DFD elongation started, the elongation of the distance to the optic disc could be compensated by a straightening of the course of the nerve fibers. If however the retinal nerve fibers were located in the center of the papillo-macular bundle and had a straight course to the optic disc already before an enlargement of gamma zone started, the fibers could not compensate the elongated DFD by straightening their course. One may assume that they get stretched in that situation. Such a stretching of papillo-macular nerve fibers might be associated with a damage to the nerve fibers and may lead to the development of deep central scotomas in highly myopic eyes with a large gamma zone and without myopic macular changes explaining such perimetric defects^25–27^. Fitting with that notion, a higher prevalence of myopic maculopathy and longer axial length was associated with a thinner peripapillary retinal nerve fiber layer in the recent Ural Eye and Medical Study, after adjusting for the prevalence and amount of glaucomatous optic neuropathy or after excluding eyes with glaucomatous optic neuropathy from the statistical analysis^28^. In a parallel manner, Omoto and colleagues reported that in 138 healthy eyes without any known eye disease the macular ganglion cell-inner plexiform layer thickness decreased significantly with narrowing of the peripapillary retinal artery angle (or angle kappa)^29^.

Narrowing of the angle kappa was strongly correlated with an elongation of the DFD. One may discuss that a posterior shift of the fovea in association with the elongation of the disc-fovea distance at the nasal side of the fovea would imply the development of a secondary divergent pseudo strabismus in myopic eyes, unless the posterior pole with the fovea is shifted backwards evenly in all meridians. Since the majority of myopic eyes does not develop a pseudo-strabismus, one may infer that indeed the foveal backward shift occurs in all meridians. It would fit with the notion of a circular enlargement of BM in the equatorial region of the globe^14^.

The inner limiting membrane is, besides the retinal nerve fibers and the large retinal vessels, the only other structure connecting the retina with the optic disc. As the retinal nerve fibers, also the inner limiting membrane in the papillo-macular region may get stretched in eyes with an elongated DFD and enlarged gamma zone. The inner limiting membrane as the basal membrane of the Müller-cells is assumed to have a relatively low elasticity, as may also be the case with the lens capsule as basal membrane of the lens epithelium or Descemet’s membrane as the basal membrane of the corneal endothelium cells. If the DFD elongates more than the low elasticity of the inner limiting membrane allows, the inner limiting membrane may get elevated to shorten the elongated distance to the optic disc. It may hold true in particular in eyes with a scleral staphyloma at the posterior pole. The phenomenon may be similar to the effect of a strained washing line. One may discuss whether such a pulling force exerted on the inner limiting membrane in highly myopic eyes with a large gamma zone may be forwarded to the deeper retinal layers, leading to an increase in the inner-retinal strain with the sequel of a macular schisis^30^. Peeling and removal of the inner limiting membrane in such a situation may then release the strain from the deeper retinal layers and might lead to a decrease in the height of the macular schisis. It may however increase the strain on the retinal nerve fibers which then are the only remaining structure (except for the large retinal vessels) connecting the macular retina with the optic disc.

Interestingly, 9 (10.5%) out of the 86 non-highly eyes showed a DFD elongation. A similar result was reported in a previous study by Yanagisawa and colleagues who observed 125 eyes of 72 patients (mean refractive error: − 3.5 ± 3.1 diopters (− 13.0 to + 3.25 diopters) with primary open-angle glaucoma for a mean follow-up time of about 5 years and who found that an increase in axial length (mean: 0.035 ± 0.10 mm) was significantly related with a lower axial length at baseline^31^. Therefore, the DFD elongation was observed not only in eyes with longer axial length but also in eyes with shorter axial length. A longer axial length is associated with a longer DFD. The reasons for the elongation of the DFD in our non-highly myopic study population may have been that the subgroup of non-highly myopic eyes with DFD elongation as compared to the non-highly myopic eyes without DFD elongation was significantly (*P* < 0.001) more myopic, and a partially arbitrary cut-off value of − 6 diopters for the definition of high myopia. It may indicate that some eyes of adults with a myopic refractive error of less than − 6 diopters may also undergo further myopic changes at their posterior pole.

The observations made in our study may have some clinical meaning. The potential stretching of the retinal nerve fibers in the papillo-macular bundle area, parallel to straightening of the retinal vessels may indicate the presence of paracentral scotomas in highly myopic eyes without that myopic macular changes such as macular BM defect might explain such scotomas. The elongation of the DFD in myopic eyes may have the psychophysical equivalent of an increased distance between the visual center and blind spot in perimetry. It may influence the morphologic-functional relationship in optic neuropathies such as the glaucomas. The enlarged gamma zone in myopic eyes is the equivalent of an enlargement of the blind spot in myopic eyes^32,33^. The decrease in angle kappa with longer axial length may lead to an accompanying change in the position in the peaks of the peripapillary retinal nerve fiber layer profile, so that in an OCT-based assessment of the optic nerve the peaks, despite a normal volume of the retinal nerve fiber layer, come to lie outside of the normal position and may be marked as abnormal or pathological.

When discussing the findings of our study, its limitations should be taken into account. First, only 89 (43.6%) out of 204 highly myopic eyes which were examined in the survey in 2001 were included into the present study. This figure of re-participation of 44% in a 10-year follow-up study roughly compares with the 5-year follow participation rate of 68.7% in the Blue Mountains Eye Study on the re-examination of eyes with myopic retinopathy^34^. As in any population-based study, however, the non-participation might have influenced the assessment of the prevalence of a DFD elongation within the study population. It may however not have had an effect on the main result, that the DFD elongation is mostly due to an enlargement of gamma zone and that it is associated with a decrease in angle kappa and a straightening of the papillomacular retinal vessels. Second, OCT images were not obtained at the baseline examination in 2001. The assessment of the OCT images taken in 2011 allowed however the identification of gamma zone and of the location of the fovea. Third, we measured the DFD on two-dimensional fundus images of the curved posterior ocular segment. It led to an underestimation of the real distance between the optic disc and the fovea. Although this principal measurement error was present in all eyes, it was more marked in eyes with a more profoundly curved posterior segment, in particular in eyes with a posterior staphyloma. Fourth, as determined by Littmann and others, the magnification of images of elements of the posterior fundus depends on the axial length^35^. Any comparison in the DFD or gamma zone width between eyes and between individuals would have necessitated the inclusion of the axial length into the assessment and comparison of the parameters. In our study however, we performed an intra-eye comparison of the DFD and other morphologic variables between images taken in 2001 and obtained in 2011. Although the axial length will have increased in a substantial number of eyes of the highly myopic group, this increase, according to data from previous studies, may have been relatively low, so that the change in axial length between 2001 and 2011, in agreement with the calculations by Littmann, may not have markedly influenced the image magnification by the optical system of the eye^35–37^. Fifth, the study participants were born in 1961 or earlier, so that the type of high myopia examined in our study may differ from the type of “school children myopia” common in the young generation today. It has therefore remained elusive whether the findings obtained in this study can be transferred onto the young highly myopic individuals of today. Sixth, future studies may address whether the findings made in our Chinese population can be transferred on population of different ethnicity.

In conclusion, the prevalence of a DFD elongation in the highly myopic eyes of our population-based sample was relatively high with a value of 64.8% during a 10-year follow-up. The DFD elongation was due to an enlargement of gamma zone, while the length of BM measured on the disc-fovea line and as distance between the temporal arterial arcades remained constant. These findings support the notion of a shifting, but otherwise stable BM at the posterior pole in axially elongated and elongating eyes. The straightening of the papillo-macular vessels with increasing gamma zone may suggest a stretching of the papillo-macular retinal nerve fibers in highly myopic eyes, potentially leading to a non-glaucomatous optic nerve damage. It may also indicate a stretching of the inner limiting membrane in the papillo-macular region.
